# Timely diagnosis and treatment of neonatal alloimmune thrombocytopenia caused by anti HPA-3a antibody: A case report

**DOI:** 10.1097/MD.0000000000015440

**Published:** 2019-05-13

**Authors:** Qiankun Yang, Xianping Lv, Yongkui Kong, Xin Liu, Ming Shao, Yanteng Zhao, Namei Xia, Shuya Wang, Huidong Li

**Affiliations:** a Department of Blood Transfusion; b Department of Surgical Clinic, The First Affiliated Hospital of Zhengzhou University, Zhengzhou, Henan, China.

**Keywords:** alloimmune, HPA-3a, neonatal, thrombocytopenia

## Abstract

**Rationale:**

Neonatal alloimmune thrombocytopenia (NAIT) caused by anti HPA-3a antibody is rare, and the clinical features of the syndrome are not specific.

**Patient concerns:**

A male infant was noted to be irritable and physical examination revealed the presence of petechiae and bruising on the right arm and thigh after born.

**Diagnoses:**

Platelet antibodies were investigated using the monoclonal antibody-specific immobilization of platelet antigens (MAIPA) assay, platelet genotyping (HPA 1–17) was performed by polymerase chain reaction technique with sequence-specific primers (PCR-SSP). The HPA genotype of the newborn was HPA-3a/b, while that of his mother and his father were HPA-3b/b and HPA-3a/a, respectively. The sera of newborn contained antibody against the platelet of newborn's father. The HPA antibody of the newborn was identified as anti HPA-3a. The newborn was confirmed as a patient of NAIT caused by anti HPA-3a antibody.

**Interventions:**

A single dose of intravenous immunoglobulin (IVIG) 1 g/kg was administered from day 3 to day 7.

**Outcomes:**

At follow-up 3 months after discharge from the hospital, the baby was developing normally and had a normal platelet count (361 × 109/L).

**Lessons:**

NAIT caused by anti HPA-3a antibody is rare, and we believe this study can provide insights for diagnosing prospective cases. Prognosis of NAIT caused by HPA3a seems to be favorable if diagnosed and treated in a timely manner.

## 1 Introduction

Neonatal alloimmune thrombocytopenia (NAIT) is the rare reason of platelet destruction, caused by maternal immunoglobulin G (IgG) alloantibodies directed against antigens on fetal or neonatal platelets.^[1]^ It rarely occurs in approximately 0.1% newborns.^[2,3]^ Clinical manifestation varies from asymptomatic thrombocytopenia to severe intracranial hemorrhage.^[4]^ There is a reported increasing mortality in NAIT, which up to 10% of affected newborns, while approximately 10% to 20% have the symptom of intracranial hemorrhage which suffer varying degrees of neurologic impairment.^[5–7]^

In clinical, several human platelet antigen (HPA) have been identified.^[8]^ Most of them are biallelic, with the high frequency antigen being defined as the “a” antigen and the low-frequency antigen as the “b” antigen. HPA-1a is the most clinically relevant platelet antigen in Caucasians, with anti-HPA-1a alloimmunization in HPA-1b homozygous mothers, which have accounted for approximately 85% of cases of NAIT.^[4]^ An additional 10% to 15% of cases are caused by HPA-5b antibodies.^[4]^ NAIT due to other platelet antigen incompatibilities is relatively uncommon. Here we present a rare case of NAIT caused by maternal HPA-3a alloimmunization.

## 2 Case presentation

This study was approved by the Ethics Committee and institutional review board of the First Affiliated Hospital of Zhengzhou University, which is registered as number FAHZU050422. Written informed consent was obtained from the patient for publication of this report.

**Figure 1:**
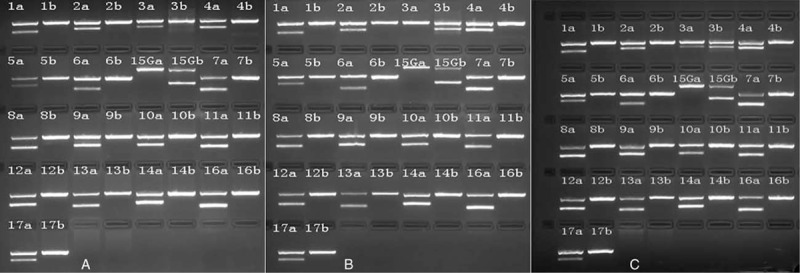
Platelet specific antigen of infant's father (A), infant's mother (B), and infant (C) by PCR-SSP. PCR-SSP = polymerase chain reaction technique with sequence-specific primers.

A 30-year-old mother gave birth to her first child by vagina after an uneventful pregnancy. She had no birth and no pregnancy before with normal platelet count and leucocytes level. She had no relative medications taking history during her pregnancy, had no history of blood transfusion, and had no hepatitis B infection. The male infant (birth weight: 4050 g) was generally healthy at birth, with Apgar scores of 9, 9, and 10 at 1, 5, and 10 minutes, respectively. Approximately 36 hours after born, the infant was noted to be irritable and physical examination revealed the presence of petechiae and bruising on the right arm and thigh, extending to the back, and to the right shoulder region. The infant's platelet count was 23 × 109/L, hemoglobin 15.9 g/dL, activated partial thromboplastin time (APTT) 36 seconds (control 26–32 seconds), and international normalized ratio (INR) 1.4. Red blood cells and white blood cell counts were in the normal range. There was no evidence of infection, malformation, hemangioma, or hepatosplenomegaly. The maternal platelet count was in the normal range and there was no familial history of bleeding disorders. Blood cultures of the infant were negative. Serum samples of the infant and the patients were tested for platelet-reactive antibodies. Platelet antibodies were investigated using the monoclonal antibody-specific immobilization of platelet antigens (MAIPA) assay previously described.^[9]^ Platelet genotyping (HPA 1–17) was performed by polymerase chain reaction technique with sequence-specific primers (PCR-SSP).^[10]^ A feto-maternal mismatch for HPA-3a was revealed (father HPA-1a/b, -2a/a, -3a/a, -4a/a, -5a/a, -6a/a, -7a/a, -8a/a, -9a/a, -10a/a, -11a/a, -12a/a, -13a/a, -14a/a, -15a/a, -16a/a, -17a/a; mother HPA-1a/ b, -2a/a, -3b/b, -4a/a, -5a/a, -6a/a, -7a/a, -8a/a, -9a/a, -10a/a, -11a/a, -12a/a, -13a/a, -14a/a, -15a/a, -16a/a, -17a/a; newborn HPA-1a/b, -2a/a, -3a/b, -4a/a, -5a/a, -6a/a, -7a/a, -8a/a, -9a/a, -10a/a, -11a/a, -12a/a, -13a/a, -14a/a, -15a/a, -16a/a, -17a/a). These results were consistent with a diagnosis of NAIT due to maternal HPA-3a antibodies (Fig. 1). Human leukocyte antigen (HLA) antibodies class-I were detectable in both the serum sample obtained after delivery, using the PakPlus kit (GTI, Waukesha, WI, USA). The infant's serum bound both IgG and IgM to the surface of platelets in the immunofluorescence test. While weakly reactive HPA-3a antibodies were identified by the MAIPA assay in the immediate postpartum serum, in addition to HLA class-I antibodies. A clinical diagnosis of NAIT caused by HAP-3a was confirmed after laboratory examination and a single dose of intravenous immunoglobulin (IVIG) 1 g/kg was administered from day 3 to day 7 with a good benefit. At discharge on day 9, the infant's platelet count was 138 × 10^9^/L. The infant's serum bound both IgG and IgM to the surface of platelets in the immunofluorescence test. While weakly reactive HPA-3a antibodies were identified by the MAIPA assay in the immediate postpartum serum, in addition to HLA class- I antibodies. At follow-up 3 months after discharge from the hospital, the baby was developing normally and had a normal platelet count (361 × 10^9^/L).

## 3 Discussion

NAIT occurs rarely in approximately 1 in 1000 to 2000 pregnancies.^[11]^ The clinical feature of the syndrome is heterogeneous, which varies from mild to severe thrombocytopenia, leading to lifelong sequelae and even death in some severe cases. Generally, newborn with NAIT has an excellent prognosis in the absence of intracerebral bleeding cases, because NAIT is a self-limiting and transient disorder. NAIT caused by anti-HPA-1a seems to occur in first pregnancy in approximately 8% of all cases.^[12]^

Antibodies against HPA-1a are the major etiological factor (about 75%) of NAIT in Caucasians, followed by HPA-5b and HPA-15. Moreover, alloantibodies against HPA-3a account for approximately 3% of all NAIT cases.^[13]^ HPA-3a antigen is located on the αIIb (GPIIb) subunit of αIIbb3 integrin (GPIIb-IIIa), which has a frequency of 85% in Caucasians. Allele frequencies of HPA-3a and HPA-3b are 0.61 and 0.38, respectively.^[14]^ HPA-3 system may be a significant cause of NAIT. Antibodies to the HPA-3a antigen system were originally described in 1980.^[15]^ Only a fraction of cases has been reported.^[16–20]^ Thus, HPA-3a would appear to be significantly immunogenic factor only less than HPA-1a and HPA-5b. With the development of technology, numerous new approaches provide highly sensitive and specific, multiplex bead-based assay for detecting human platelet antibodies,^[21–23]^ which can contribute to a high diagnostic rate of the NAIT.

Our report on a newborn with severe thrombocytopenia caused by HPA-3a antibodies confirms the significance of timely diagnosis on alloimmunization against HPA-3a. On the cases of thrombocytopenia which are mainly caused by HPA-3a antibodies, intravenous immunoglobulin treatment may be the best choice for neonates with immune thrombocytopenia.^[24]^ The mechanism of action of IVIG has been assumed as competitive inhibition of antibody binding or reticuloendothelial blockage.^[25]^ As for our patient, the treatment with IVIG appeared to be effective after 4 days infusion due to the timely diagnosis on the newborn, which proved the significance of early diagnosis and treatment on NAIT.

The incidence of NAIT is very low because of maternal-fetal platelet antigen mismatches. This could be caused by the fact that many newborns who acquire these alloantibodies only few have severe thrombocytopenia, and this leads to the disease being undiagnosed or undetected. Therefore, the timely and early detection and diagnosis of these alloantibodies is of prime importance for applying appropriate therapy as soon as possible and decreasing the incidence of sequelae as far as we can.

## 4 Conclusion

This case highlights the clinical significance of HPA-3a, even though potential difficulties on diagnosis maybe encountered in the detection of platelet-specific antibodies. In clinical, timely diagnosis and treatment for NAITP newborn caused by anti HPA-3a antibody can improve the prognosis. It also could provide successful experiences and references for the similar cases.

## Author contributions

**Data curation:** Xin Liu.

**Formal analysis:** Yongkui Kong.

**Investigation:** Qiankun Yang.

**Methodology:** Xianping Lv.

**Software:** Yanteng Zhao.

**Writing – original draft:** Ming Shao, Namei Xia, Shuya Wang.

**Writing – review & editing:** Huidong Li.
